# Association of ultra-processed food consumption with cardiovascular mortality in the US population: long-term results from a large prospective multicenter study

**DOI:** 10.1186/s12966-021-01081-3

**Published:** 2021-02-03

**Authors:** Guo-Chao Zhong, Hai-Tao Gu, Yang Peng, Kang Wang, You-Qi-Le Wu, Tian-Yang Hu, Feng-Chuang Jing, Fa-Bao Hao

**Affiliations:** 1 Department of Hepatobiliary Surgery, the Second Affiliated Hospital of Chongqing Medical University, Chongqing, China; 2 Department of Gastrointestinal Surgery, the Second Affiliated Hospital of Chongqing Medical University, Chongqing, China; 3 Department of Geriatrics, the Fifth People’s Hospital of Chengdu, Chengdu, China; 4 Department of Endocrine and Breast Surgery, the First Affiliated Hospital of Chongqing Medical University, Chongqing, China; 5 Department of Nutrition and Food Hygiene, School of Public Health and Management, Chongqing Medical University, Chongqing, China; 6 Department of Cardiology, the Second Affiliated Hospital of Chongqing Medical University, Chongqing, China; 7 Department of Cardiovascular Medicine, Children’s Hospital of Chongqing Medical University, Chongqing, China; 8 Department of Neurosurgery, Qingdao Women and Children’s Hospital, Qingdao University, Qingdao, Shandong China

**Keywords:** Ultra-processed food, Cardiovascular mortality, Prospective study, Risk factor

## Abstract

**Background:**

Ultra-processed foods have now become dominant in the global food system. Whether their consumption is associated with cardiovascular mortality remains controversial. Moreover, data on ultra-processed foods and cardiovascular outcomes are scarce in the US population. We aimed to examine the association of ultra-processed food consumption with cardiovascular mortality in a US population.

**Methods:**

A population-based cohort of 91,891 participants was identified from the Prostate, Lung, Colorectal, and Ovarian Cancer Screening Trial. Dietary data were collected through a validated 137-item food frequency questionnaire. Ultra-processed foods were defined by the NOVA classification. Cox regression was used to calculate hazard ratios (HRs) and 95% confidence intervals (CIs) for cardiovascular mortality. Restricted cubic spline regression was used to test nonlinearity. Subgroup analyses were conducted to identify the potential effect modifiers.

**Results:**

After an average follow-up of 13.5 years (1,236,049.2 person-years), 5490 cardiovascular deaths were documented, including 3985 heart disease deaths and 1126 cerebrovascular deaths. In the fully adjusted model, participants in the highest vs. the lowest quintiles of ultra-processed food consumption had higher risks of death from cardiovascular disease (HR_quintile 5 vs. 1_, 1.50; 95% CI, 1.36–1.64) and heart disease (HR_quintile 5 vs. 1_, 1.68; 95% CI, 1.50–1.87) but not cerebrovascular disease (HR_quintile 5 vs. 1_, 0.94; 95% CI, 0.76–1.17). A nonlinear dose–response pattern was observed for overall cardiovascular and heart disease mortality (all *P*_nonlinearity_ < 0.05), with a threshold effect observed at ultra-processed food consumption of 2.4 servings/day and 2.3 servings/day, respectively; below the thresholds, no significant associations were observed for these two outcomes. Subgroup analyses showed that the increased risks of mortality from ultra-processed foods were significantly higher in women than in men (all *P*_interaction_ < 0.05).

**Conclusions:**

High consumption of ultra-processed foods is associated with increased risks of overall cardiovascular and heart disease mortality. These harmful associations may be more pronounced in women. Our findings need to be confirmed in other populations and settings.

**Supplementary Information:**

The online version contains supplementary material available at 10.1186/s12966-021-01081-3.

## Background

Cardiovascular disease (CVD) is the most common cause of death in the US and worldwide, with an estimated 0.84 million and 17.90 million cardiovascular deaths in 2016, respectively [1, 2]. The American Heart Association has released the 2030 Impact Goal for improving cardiovascular health and preventing CVD, and one of approaches achieving this goal may be through targeting modifiable CVD risk factors [3]. It is now well known that diet can directly and strongly affect the occurrence and development of CVD [4, 5].

Ultra-processed foods are industrial formulations mostly or entirely made from substances derived from additives and foods, with little or even no whole foods [6]. They are usually ready-to-eat, highly affordable, hyper-palatable, and energy-dense, and are marketed intensively and packaged attractively. Ultra-processed foods have now become dominant in the global food system [7]. In the US, the percentage of energy from ultra-processed foods has reached as high as 58.5% in the period 2007–2012 [8].

Several observational studies have showed that higher consumption of ultra-processed foods is associated with higher incidences of coronary heart and cerebrovascular diseases [9] as well as CVD risk factors (hypertension, type 2 diabetes, and obesity) [10–12]. However, whether ultra-processed food consumption is a predictor of cardiovascular mortality remains controversial. Specifically, modelling studies showed that decreasing consumption of ultra-processed foods was associated with a reduced risk of cardiovascular mortality [13, 14], whereas observational studies on this subject showed a null association in a cohort of 19,899 Spanish university graduates aged 20–91 years [15] and a nationally representative cohort of 11,898 American adults aged ≥20 years [16]. Importantly, these two observational studies observed limited cardiovascular deaths, thus a significant association between ultra-processed food consumption and cardiovascular mortality could have been missed due to insufficient power. Additionally, to our knowledge, the above-mentioned study (ref. [16]) is the only one investigating the association of ultra-processed food consumption with cardiovascular outcomes in the US population to date.

Considering the need for data from large studies on this topic in the US population, we performed a prospective multicenter study of 91,891 American adults with long-term follow-up to examine the association of ultra-processed food consumption with cardiovascular mortality.

## Methods

We reported this study in accordance with the Strengthening the Reporting of Observational Studies in Epidemiology statement.

### Study population

Between November 1993 and September 2001, nearly 155,000 American adults aged 55 to 74 years were enrolled to the Prostate, Lung, Colorectal, and Ovarian (PLCO) Cancer Screening Trial in ten screening centers (Washington, Pittsburgh, Honolulu, Denver, Marshfield, Minneapolis, Birmingham, Salt Lake City, Detroit, and St Louis) across the US, which is a randomized controlled trial for investigating whether screening for prostate, lung, colorectal, and ovarian cancer could decrease the risk of mortality from these cancers. Study design of this trial has been reported elsewhere [17]. The PLCO Cancer Screening Trial was approved by the Institutional Review Boards of the US National Cancer Institute and each recruitment center. All participants provided written informed consent.

**Fig. 1:**
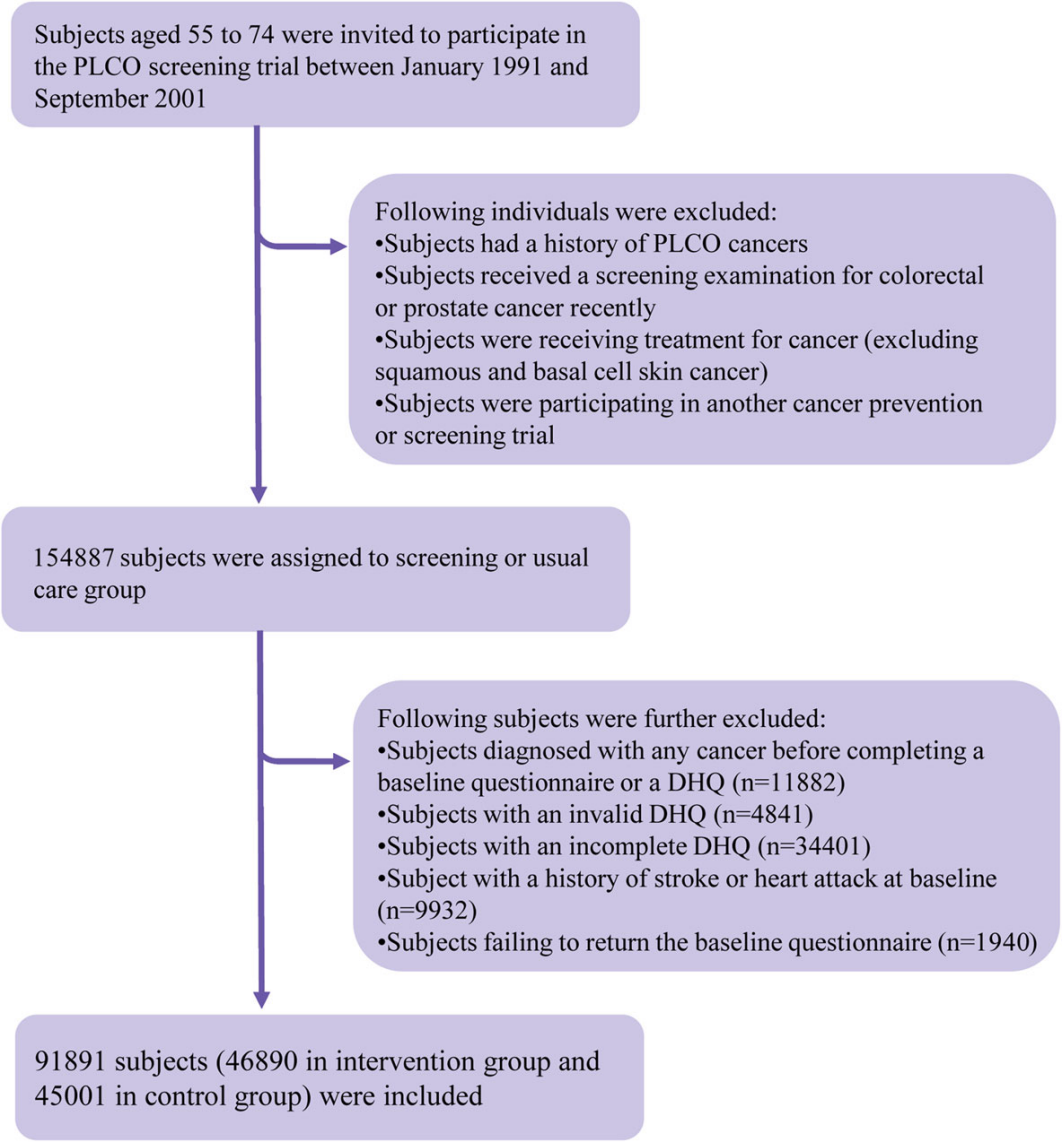
The study flow chart of identifying eligible subjects. PLCO, Prostate, Lung, Colorectal, and Ovarian; DHQ, diet history questionnaire

**Fig. 2:**
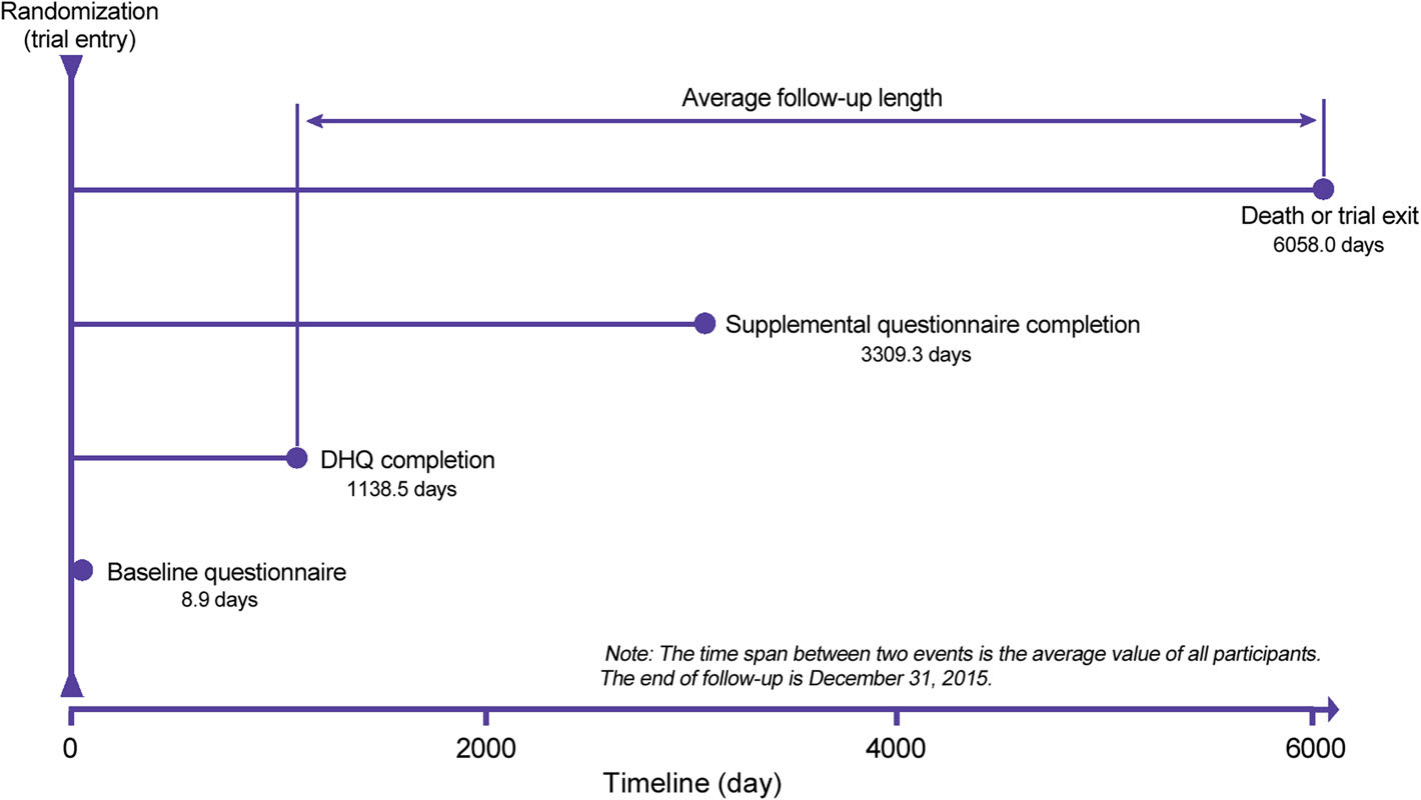
The timeline and follow-up scheme of the present study. DHQ, diet history questionnaire

The following subjects were excluded in the present study: (1) subjects receiving a diagnosis of any cancer before completing a baseline questionnaire or a diet history questionnaire (DHQ; *n* = 11,882); (2) subjects with an invalid DHQ, which is defined as the presence of extreme values of energy intake (i.e., the first or last percentile), death date prior to DHQ completion date, missing date of DHQ completion, or ≥ 8 missing DHQ items (*n* = 4841); (3) subjects failing to complete a DHQ (*n* = 34,401); (4) subject with a history of stroke or heart attack at baseline (*n* = 9932); and (5) subjects failing to return the baseline questionnaire (*n* = 1940). After exclusions, a total of 91,891 subjects were included (Fig. 1). Of note, a comparison of baseline characteristics of included (*n* = 91,891) and excluded (*n* = 62,996) subjects showed that there were no marked differences in age, sex, race, educational level, body mass index (BMI), smoking status, history of diabetes, and history of hypertension between two groups, suggesting that the potential for nonparticipation biases was low in our study. For all eligible subjects, follow-up time was calculated from the date of DHQ completion to the date of death, study dropout, or the end of follow-up (i.e., December 31, 2015), whichever came first (Fig. 2).

### Data collection

Baseline data, including sex, marital status, race, height, body weight, educational level, history of diabetes or hypertension, and smoking status, were collected with a self-administrated baseline questionnaire. BMI was calculated as body weight (kg) divided by height squared (m^2^). Age at DHQ completion, alcohol intake, and food consumption were collected with a DHQ (version 1.0, National Cancer Institute, 2007). The DHQ is a self-administered 137-item food frequency questionnaire, which is designed to assess the frequency and portion size of food consumption and nutrient intake during the past year. The Eating at America’s Table Study had validated the DHQ performance in a nationally representative sample of 1640 subjects against four 24-h dietary recalls, indicating that the DHQ had good performance in the estimation of dietary intake [18]. Daily consumption of each food in the DHQ was estimated by multiplying food frequency by portion size; dietary intake of nutrients and energy were calculated by the DietCalc software [19], which mainly considered food frequency and portion size and used nutrient values from the USDA’s 1994–96 Continuing Survey of Food Intakes by Individuals [20] or the Nutrition Data Systems for Research [21]. Healthy Eating Index-2005, a measure of diet quality, was calculated using the method described in the literature [22]. Physical activity level was estimated based on the frequency and duration of moderate and strenuous activities that were collected with a self-administrated supplemental questionnaire.

### Assessment of ultra-processed food consumption

Two dietitians classified all food and drink items of the DHQ into one of the four food groups defined by the NOVA classification [6]. Based on the purpose, nature, and degree of food processing, the NOVA classification outlines four food groups: unprocessed or minimally processed foods, processed culinary ingredients, processed foods, and ultra-processed foods. The detailed description, including definition and example, for each group is available elsewhere [6]. In the present study, we focused on ultra-processed foods, which include sour cream, cream cheese, ice cream, frozen yogurt, fried foods, breads, cookies, cakes, pastries, salty snacks, breakfast cereals, instant noodles and soups, sauces, margarine, candy, soft drinks, fruit drinks, restaurant/industrial hamburgers, hot dogs, and pizza. Based on a reported categorization method [23], all ultra-processed foods were further categorized into nine food groups for relevant analyses, namely soft drinks, cereals, ultra-processed fruits and vegetables, ultra-processed dairy products, meat and fish, sauces and dressings, salty snacks, sugary products, and margarine. Table S1 shows the full list of ultra-processed foods in each food group.

The amount consumed of each food item (65 items, see Table S1) was summed together to calculate an individual’s overall consumption of ultra-processed foods. The energy provided by each food was estimated by dividing the amount consumed in grams by 100 and then multiplying the corresponding energy value (kcal) per 100 g of food (Table S1), which was from the USDA Food and Nutrient Database for Dietary Studies 2015–2016 [24]. The estimated enegy value of each food item was then summed together to calculate total energy from ultra-processed foods. Importantly, ultra-processed food consumption used for all analyses was adjusted for energy intake from diet using the residual method for removing extraneous variation of ultra-processed food consumption due to energy intake [25].

### Outcome assessment

Vital status was ascertained primarily through a mailed annual study update form. Individuals who did not return the form were contacted repeatedly via telephone or e-mail. Additionally, information on vital status was supplemented by periodic linkage to the US National Death Index to increase its completeness. The *International Classification of Diseases, ninth Revision (ICD-9)* was used to define the underlying causes of mortality obtained from death certificates: CVD (codes: 390–459), heart disease (codes 390–398, 402, 404, and 410–429), and cerebrovascular disease (codes 430–438).

### Statistical analysis

As there were seven covariates with missing data (see Table S2), for increasing statistical power and reducing potential biases, multiple imputation by chained equations was applied to impute missing data (the number of imputations = 25) [26], with the assumption that the above-mentioned data were missing at random. All variables involved in statistical analyses were employed to yield the imputed data sets. Main analyses were repeated in participants with complete data for comparison.

Cox proportional hazards regression was applied to estimate hazard ratios (HRs) and 95% confidence intervals (CIs) for the association between ultra-processed food consumption and cardiovascular mortality, with follow-up time as the underlying time metric. Ultra-processed food consumption was divided into quintiles, with the lowest quintile as reference group. To test linear trends in risk estimates across quintiles of energy-adjusted ultra-processed food consumption, the median value of each quintile was first assigned to each participant in the quintile to yield an ordinal variable. This ordinal variable was then entered as a continuous variable in regression models, and its *P* value, which was obtained with the Wald test, was used to indicate the significance of linear trends. In multivariable analyses, covariate selection was based on the change-in-estimate approach [27] and the existing literature. Specifically, model 1 was adjusted for age, sex, race, educational level, marital status, and study center; model 2 was further adjusted for aspirin use, history of hypertension, history of diabetes, smoking status, alcohol consumption, BMI, physical activity level, and energy intake from diet. To assess how robust our results were to the potential unmeasured confounding, we calculated the E-value through an online calculator (https://mmathur.shinyapps.io/evalue/) [28], with an assumption of outcome prevelence less than 15%. The E-value represents what the minimum HR would have to be for an unmeasured confounder, conditional on the measured covariates, to negate the observed association of ultra-processed food consumption with cardiovascular mortality. No violation of the proportional hazards assumption was found using Schoenfeld residuals method (all *P* > 0.05). We expressed ultra-processed food consumption in all main analyses as daily servings mainly based on the USDA Pyramid Servings Database [29]. Meanwhile, we expressed ultra-processed food consumption as serving per day/kilogram body weight in supplementary analyses to examine the potential impacts of body size. For comparison with the published data, we also tested the association between the proportion of energy from ultra-processed foods to total daily energy intake (% energy) and cardiovascular mortality.

Prespecified subgroup analyses were conducted to assess whether the observed association of ultra-processed food consumption with cardiovascular mortality was modified by age (≥65 vs. < 65 years), sex (male vs. female), BMI (≥25 vs. < 25 kg/m^2^), smoking status (current or former vs. never), and alcohol consumption (no, light, or moderate vs. heavy). Here, light, moderate, and heavy alcohol consumption were defined as ≤6 g/day, > 6–28 g/day for male and > 6–14 g/day for female, and > 28 g/day for male and > 14 g/day for female, respectively [30]. A *P*_interaction_ was obtained through a likelihood ratio test, which compares the models with and without interaction terms.

Restricted cubic spline regression [31] with four knots at the 5th, 35th, 65th, and 95th percentiles was used to explore the potential dose–response relationship between ultra-processed consumption and cardiovascular mortality. The reference level was set at 0 serving/day. A *P*_nonlinearity_ was obtained by testing the null hypothesis that regression coefficients of the second and third splines are equal to zero [31].

Sensitivity analyses were performed to evaluate the robustness of our results: (1) excluding deaths occurring within the first five years of follow-up to determine the potential effects of reverse causation; (2) excluding subjects with extreme values of energy intake, which are defined as < 800 or > 4000 kcal/day and < 500 or > 3500 kcal/day for men and women, respectively [32]; (3) including subjects with history of cancer at baseline; (4) including subjects with history of heart attack or stroke at baseline; (5) repeating main analyses with competing risk regression [33] to assess the potential effects of competing risk bias, with non-CVD causes of death as competing events; (6) adjustment for propensity score on unadjusted model (all covariates in model 2 were used to calculate propensity score); and (7) additional adjustment for several indicators of diet quality, including Healthy Eating Index-2005, intakes of sodium, added sugars, and saturated fatty acids, and consumption of red meat, processed meat, whole grain, fruit, vegetable, dietary fiber, and dairy.

We calculated the proportion of each food group in total energy-adjusted serving size or total energy of ultra-processed foods to quantify their contributions to ultra-processed food consumption. In addition, we tested the association between ultra-processed food consumption by food group and cardiovascular mortality. To validate our study design and methods, we used all-cause mortality as a positive control outcome, given the well-established association of ultra-processed food consumption with all-cause mortality [15, 16, 23, 34]. The statistical significance level was set at *P* < 0.05 under a two-tailed test. Statistical analyses were performed using STATA version 12.0 (StataCorp, College Station, TX).

## Results

### Participant characteristics

In the entire study population, the average (standard deviation) energy-adjusted consumption of ultra-processed foods was 2.7 (3.8) servings/day; the average (standard deviation) energy contribution of ultra-processed foods in the diet was 35.5% (16.6%). Participants in the highest vs. the lowest quintiles of ultra-processed food consumption were younger and more likely to be male, married, non-Hispanic white, current or former smokers, and overweight or obesity, were more likely to have a history of diabetes or hypertension, had lower levels of alcohol consumption, physical activity, and education but higher energy intake from diet, and had lower Healthy Eating Index-2005 (all *P*_trend_ < 0.001) (Table 1). Compared with participants in the lowest quintile of ultra-processed food consumption, those in the highest quintile had higher consumption of red meat and dairy but lower consumption of fruit, and higher intakes of cholesterol, saturated fatty acids, polyunsaturated fatty acids, carbohydrate, fat, protein, added sugar, sodium, magnesium, potassium, and calcium (all *P*_trend_ < 0.001) (Table 1).

**Table 1:** Baseline characteristics of study population according to energy-adjusted ultra-processed food consumption in 91,891 participants

Characteristics	Quintiles of energy-adjusted ultra-processed food consumption, range (mean), servings/day					
	< 0.5 (0.1)	0.5–< 1.1 (0.8)	1.1–< 2.1 (1.6)	2.1–≤4.0 (3.0)	> 4.0 (8.2)	*P*_trend_
No. of participants	18,378	18,378	18,379	18,377	18,379	
Age (years)	66.3 ± 5.7	66.1 ± 5.7	65.6 ± 5.7	64.9 ± 5.6	63.7 ± 5.3	< 0.001
Male	5976 (32.5)	7396 (40.2)	9058 (49.3)	9592 (52.2)	10,521 (57.2)	< 0.001
Married	13,488 (73.4)	14,489 (78.8)	14,833 (80.7)	14,829 (80.7)	14,507 (78.9)	< 0.001
History of diabetes	836 (4.5)	842 (4.6)	882 (4.8)	1071 (5.8)	1693 (9.2)	< 0.001
History of hypertension	5066 (27.6)	5433 (29.6)	5451 (29.7)	5723 (31.1)	6266 (34.1)	< 0.001
Alcohol consumption (g/day)	13.7 ± 38.5	8.6 ± 19.7	8.5 ± 18.8	8.4 ± 18.8	8.6 ± 24.1	< 0.001
Energy intake from diet (kcal/day)	1440.6 ± 646.6	1516.5 ± 555.6	1722.0 ± 628.5	1880.6 ± 724.4	2123.0 ± 868.9	< 0.001
Physical activity (min/week) ^a^	133.2 ± 127.6	124.3 ± 121.7	123.2 ± 120.7	122.5 ± 123.0	123.6 ± 127.4	< 0.001
Healthy Eating Index-2005	63.9 ± 11.7	61.4 ± 10.6	59.5 ± 10.5	58.5 ± 10.5	56.7 ± 10.8	< 0.001
Race						
Non-Hispanic white	15,991 (87.0)	16,925 (92.1)	16,910 (92.0)	16,907 (92.0)	16,815 (91.5)	< 0.001
Non-Hispanic black	449 (2.4)	425 (2.3)	559 (3.0)	711 (3.9)	837 (4.6)	
Hispanic	322 (1.8)	240 (1.3)	252 (1.4)	270 (1.5)	276 (1.5)	
Others ^b^	1616 (8.8)	788 (4.3)	658 (3.6)	489 (2.7)	451 (2.5)	
Educational level						
College below	10,870 (59.1)	11,391 (62.0)	11,701 (63.7)	11,913 (64.8)	12,265 (66.7)	< 0.001
College graduate	3573 (19.4)	3437 (18.7)	3235 (17.6)	3154 (17.2)	2968 (16.1)	
Postgraduate	3935 (21.4)	3550 (19.3)	3443 (18.7)	3310 (18.0)	3146 (17.1)	
Smoking status						
Current	1432 (7.8)	1572 (8.6)	1617 (8.8)	1691 (9.2)	2099 (11.4)	< 0.001
Former	7614 (41.4)	7484 (40.7)	7563 (41.2)	7679 (41.8)	8039 (43.7)	
Never	9332 (50.8)	9322 (50.7)	9199 (50.1)	9007 (49.0)	8241 (44.8)	
Body mass index (kg/m^2^)						
< 18.5	243 (1.3)	134 (0.7)	80 (0.4)	84 (0.5)	77 (0.4)	< 0.001
18.5–24.9	8650 (47.7)	6981 (38.5)	6053 (33.4)	5365 (29.5)	4193 (23.2)	
25.0–30.0	6570 (36.2)	7591 (41.8)	8100 (44.6)	8205 (45.2)	7921 (43.8)	
> 30	2671 (14.7)	3439 (19.0)	3910 (21.6)	4503 (24.8)	5910 (32.7)	
Food consumption						
Red meat (g/day)	37.4 ± 34.1	50.0 ± 37.0	62.5 ± 46.2	71.3 ± 55.2	85.2 ± 67.5	< 0.001
Fruit (g/day)	307.1 ± 248.4	263.6 ± 191.9	261.2 ± 190.4	268.2 ± 201.1	268.1 ± 241.0	< 0.001
Vegetable (g/day)	288.9 ± 211.1	264.7 ± 167.8	275.2 ± 168.0	287.8 ± 178.6	300.1 ± 195.4	< 0.001
Dietary fiber (g/day)	17.8 ± 9.4	16.6 ± 7.6	17.7 ± 7.8	18.6 ± 8.2	19.3 ± 8.9	< 0.001
Whole grain (servings/day)	1.1 ± 0.8	1.1 ± 0.8	1.2 ± 0.8	1.2 ± 0.8	1.2 ± 0.9	< 0.001
Dairy (cups/day)	1.3 ± 1.2	1.3 ± 1.1	1.4 ± 1.1	1.4 ± 1.1	1.5 ± 1.2	< 0.001
Nutrient intake						
Cholesterol (mg/day)	154.9 ± 108.2	181.3 ± 105.1	211.8 ± 122.3	232.8 ± 137.3	263.8 ± 162.0	< 0.001
Saturated fatty acids (g/day)	14.0 ± 8.1	17.0 ± 8.4	20.4 ± 10.2	22.7 ± 12.3	25.9 ± 14.4	< 0.001
Polyunsaturated fatty acids (g/day)	11.1 ± 6.3	12.4 ± 6.0	14.3 ± 6.9	15.6 ± 7.8	17.2 ± 9.1	< 0.001
Carbohydrate (g/day)	187.5 ± 79.9	193.7 ± 70.6	216.3 ± 76.2	236.6 ± 86.3	269.6 ± 111.4	< 0.001
Fat (g/day)	46.0 ± 24.4	53.8 ± 24.5	63.7 ± 29.4	70.3 ± 34.9	79.4 ± 41.0	< 0.001
Protein (g/day)	56.2 ± 25.4	59.2 ± 24.0	66.4 ± 27.3	71.7 ± 31.2	79.0 ± 36.0	< 0.001
Added sugar (tsp/day)	7.1 ± 4.0	9.2 ± 4.4	11.7 ± 5.6	14.2 ± 7.3	19.9 ± 14.1	< 0.001
Sodium (mg/day)	2171.7 ± 934.5	2404.5 ± 919.2	2735.1 ± 1058.3	2980.1 ± 1237.3	3318.3 ± 1457.8	< 0.001
Magnesium (mg/day)	311.1 ± 134.3	299.4 ± 113.0	319.1 ± 118.8	332.9 ± 128.0	348.4 ± 138.8	< 0.001
Potassium (mg/day)	3102.0 ± 1260.0	3038.1 ± 1110.3	3228.2 ± 1163.6	3365.9 ± 1261.7	3490.7 ± 1411.7	< 0.001
Calcium (mg/day)	688.8 ± 410.3	686.2 ± 368.2	741.0 ± 380.5	783.3 ± 398.7	847.9 ± 444.4	< 0.001

Values are mean (standard deviation) or counts (percentage) as indicated

^a^ Total time of moderate to vigorous physical activities per week

^b^ “Others” refers to Asian, Pacific Islander, or American Indian

### Ultra-processed foods and cardiovascular mortality

During an average (standard deviation) follow-up of 13.5 (3.3) years (1,236,049.2 person-years), a total of 5490 cardiovascular deaths were documented, of which 3985 (72.6%) and 1126 (20.5%) were classified as deaths from heart disease and cerebrovascular disease, respectively. Crude mortality rates of CVD, heart disease, and cerebrovascular disease were 44.42, 32.24, and 9.11 per 10,000 person-years, respectively. After the full adjustment for confounders, participants in the highest vs. the lowest quintiles of ultra-processed food consumption were found to be at increased risks of overall cardiovascular (HR _quintile 5 vs. 1_, 1.50; 95% CI, 1.36–1.64; *P*_trend_ < 0.001; E-value, 2.37) and heart disease mortality (HR _quintile 5 vs. 1_, 1.68; 95% CI, 1.50–1.87; *P*_trend_ < 0.001; E-value, 2.75) (Table 2). No significant association was observed for cerebrovascular mortality (HR _quintile 5 vs. 1_, 0.94; 95% CI, 0.76–1.17; *P*_trend_ = 0.741). When the above-mentioned analyses were performed in participants with complete data, similar results were obtained (Table S3). When ultra-processed food consumption was expressed as serving per day/kilogram body weight or % energy, the initial results did not alter substantially (Tables S4 and S5).

**Table 2:** Association between energy-adjusted ultra-processed food consumption (daily serving) and cardiovascular mortality

Causes of mortality	Quintiles of energy-adjusted ultra-processed food consumption, range (mean), servings/day					*P*_trend_
	< 0.5 (0.1)	0.5–< 1.1 (0.8)	1.1–< 2.1 (1.6)	2.1–≤4.0 (3.0)	> 4.0 (8.2)	
No. of participants	18,378	18,378	18,379	18,377	18,379	
Person-years	247,441.39	247,018.70	247,436.48	247,501.44	246,651.21	
Cardiovascular disease						
No. of deaths	991	1006	1002	1135	1356	
Death rate ^a^	40.05	40.73	40.50	45.86	54.98	
Model 1 ^b^	1.00 (reference)	1.01 (0.93, 1.11)	1.01 (0.93, 1.11)	1.24 (1.14, 1.35)	1.62 (1.49, 1.76)	< 0.001
Model 2 ^c^	1.00 (reference)	1.00 (0.91, 1.09)	1.01 (0.92, 1.11)	1.20 (1.09, 1.31)	1.50 (1.36, 1.64)	< 0.001
Heart disease						
No. of deaths	670	689	683	846	1097	
Death rate ^a^	27.08	27.89	27.60	34.18	44.48	
Model 1 ^b^	1.00 (reference)	1.02 (0.92, 1.14)	1.01 (0.91, 1.13)	1.34 (1.20, 1.48)	1.87 (1.69, 2.06)	< 0.001
Model 2 ^c^	1.00 (reference)	1.01 (0.90, 1.12)	0.99 (0.89, 1.11)	1.26 (1.13, 1.41)	1.68 (1.50, 1.87)	< 0.001
Cerebrovascular disease						
No. of deaths	256	236	246	212	176	
Death rate ^a^	10.35	9.55	9.94	8.57	7.14	
Model 1 ^b^	1.00 (reference)	0.93 (0.77, 1.11)	0.99 (0.83, 1.19)	0.95 (0.79, 1.15)	0.90 (0.74, 1.10)	0.406
Model 2 ^c^	1.00 (reference)	0.91 (0.76, 1.10)	1.04 (0.86, 1.25)	1.00 (0.82, 1.21)	0.94 (0.76, 1.17)	0.741

Values are hazard ratios (95% confidence intervals)

^a^ Crude death rate per 10,000 person-years

^b^ Adjusted for age (years), sex (male, female), race (non-Hispanic white, non-Hispanic black, Hispanic, others), educational level (college below, college graduate, postgraduate), marital status (married, widowed, divorced, separated, never married), and study center (10 categories)

^c^ Adjusted for model 1 plus aspirin use (yes, no), history of hypertension (yes, no), history of diabetes (yes, no), smoking status (current, former, never), alcohol consumption (g/day), body mass index (< 18.5, 18.5–24.9, 25.0–30.0, > 30.0 kg/m^2^), physical activity (min/week), and energy intake from diet (kcal/day)

### Subgroup analyses

A significant interaction between ultra-processed food consumption and sex was detected for overall cardiovascular (*P*_interaction_ < 0.001) and heart disease mortality (*P*_interaction_ = 0.001) but not for cerebrovascular mortality (*P*_interaction_ = 0.140) (Table 3). Specifically, the highest fifth of ultra-processed food consumption was found to be associated with higher risks of death from CVD and heart disease in women (CVD: HR _quintile 5 vs. 1_, 1.93; 95% CI, 1.68–2.21; heart disease: HR _quintile 5 vs. 1_, 2.17; 95% CI, 1.84–2.55) than in men (CVD: HR _quintile 5 vs. 1_, 1.23; 95% CI, 1.08–1.40; heart disease: HR _quintile 5 vs. 1_, 1.39; 95% CI, 1.20–1.61). No significant interaction was found for remaining predefined factors.

**Table 3:** Subgroup analyses on the association between energy-adjusted ultra-processed food consumption (daily serving) and cardiovascular mortality

Subgroup variable	Overall cardiovascular mortality		Heart disease mortality		Cerebrovascular disease mortality	
	HR_quintile 5 vs. 1_ (95% CI) ^a^	*P*_interaction_	HR_quintile 5 vs. 1_ (95% CI) ^a^	*P*_interaction_	HR_quintile 5 vs. 1_ (95% CI) ^a^	*P*_interaction_
Age (years)						
≥ 60	1.47 (1.33, 1.61)	0.066	1.64 (1.47, 1.84)	0.182	0.90 (0.72, 1.13)	0.486
< 60	1.81 (1.28, 2.56)		1.89 (1.30, 2.77)		1.81 (0.70, 4.68)	
Sex						
Male	1.23 (1.08, 1.40)	< 0.001	1.39 (1.20, 1.61)	0.001	0.75 (0.56, 1.02)	0.140
Female	1.93 (1.68, 2.21)		2.17 (1.84, 2.55)		1.15 (0.85, 1.58)	
Body mass index (kg/m^2^)						
≥ 25	1.53 (1.36, 1.72)	0.814	1.68 (1.47, 1.91)	0.935	1.09 (0.83, 1.43)	0.357
< 25	1.45 (1.23, 1.70)		1.72 (1.42, 2.09)		0.69 (0.47, 1.03)	
Smoking status						
Current or former	1.44 (1.28, 1.63)	0.260	1.61 (1.40, 1.86)	0.315	0.94 (0.71, 1.24)	0.532
Never	1.61 (1.39, 1.85)		1.79 (1.52, 2.12)		0.96 (0.69, 1.34)	
Alcohol consumption (g/day) ^b^						
No, light or moderate	1.58 (1.43, 1.75)	0.144	1.75 (1.55, 1.97)	0.417	1.03 (0.82, 1.30)	0.484
Heavy	1.11 (0.86, 1.42)		1.32 (0.99, 1.76)		0.60 (0.33, 1.11)	

^a^ Adjusted for age (years), sex (male, female), race (non-Hispanic white, non-Hispanic black, Hispanic, others), educational level (college below, college graduate, postgraduate), marital status (married, widowed, divorced, separated, never married), study center (10 categories), aspirin use (yes, no), history of hypertension (yes, no), history of diabetes (yes, no), smoking status (current, former, never), alcohol consumption (g/day), body mass index (< 18.5, 18.5–24.9, 25.0–30.0, > 30.0 kg/m^2^), physical activity (min/week), and energy intake from diet (kcal/day). In subgroup analyses stratified by sex and smoking status, hazard ratios were not adjusted for the stratification factor

^b^ Light, moderate, and heavy alcohol consumption are defined as ≤6 g/day, > 6–28 g/day for male and > 6–14 g/day for female, and > 28 g/day for male and > 14 g/day for female, respectively

### Dose–response analyses

**Fig. 3:**
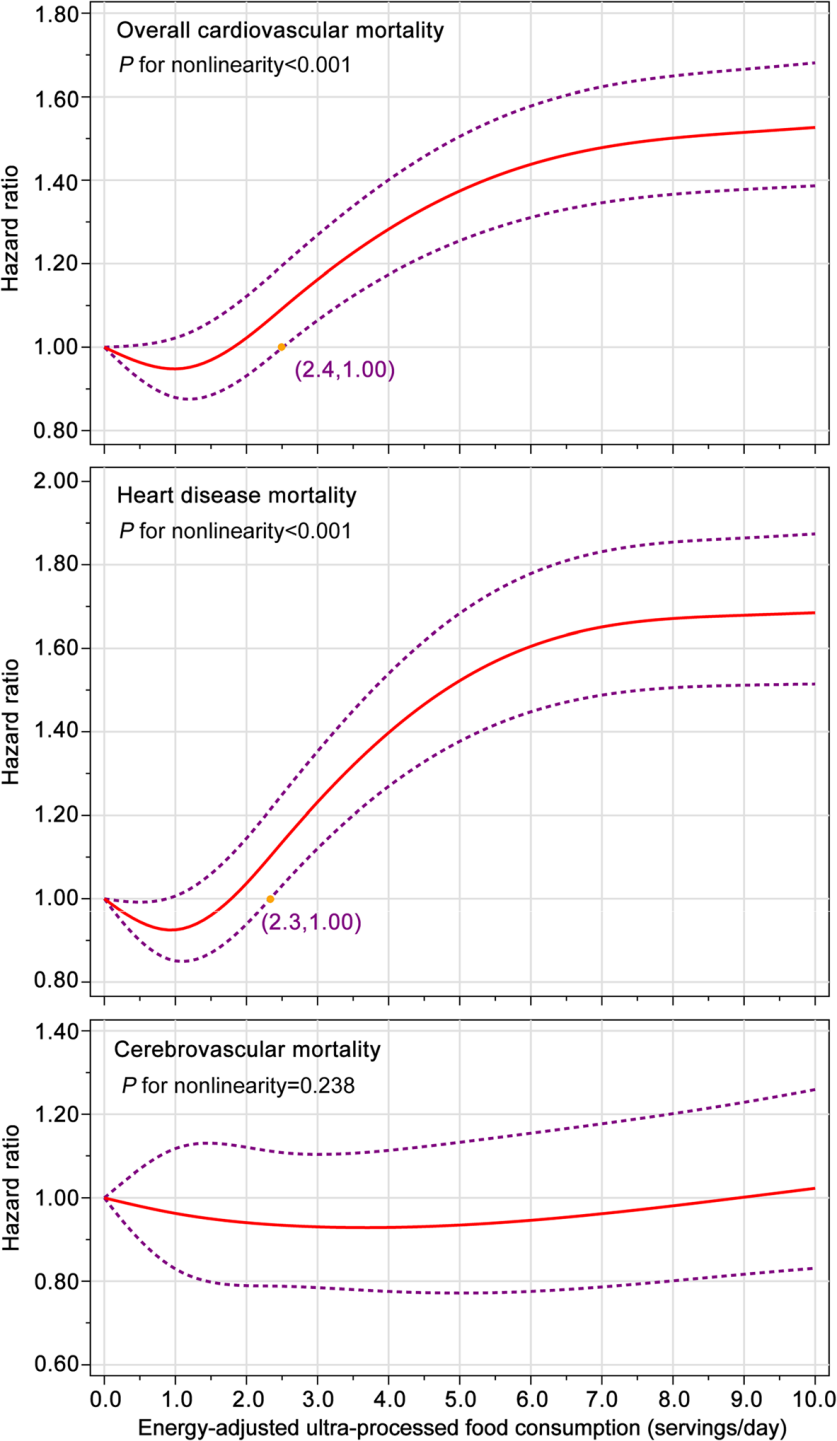
Nonlinear dose–response analyses on energy-adjusted ultra-processed food consumption and cardiovascular mortality in the whole study population. The reference level was set at 0 serving/day. Hazard ratio was adjusted for age, sex, race, educational level, marital status, study center, aspirin use, history of hypertension, history of diabetes, smoking status, alcohol consumption, body mass index, physical activity level, and energy intake from diet. The red solid line represents the fitted nonlinear trend, and the purple short-dash line represents corresponding 95% confidence interval

In the whole study population, ultra-processed food consumption was found to be associated with risks of death from CVD (*P*_nonlinearity_ < 0.001) and heart disease (*P*_nonlinearity_ < 0.001) in a nonlinear dose–response manner (Fig. 3); furthermore, a threshold effect was observed at ultra-processed food consumption of 2.4 servings/day for overall cardiovascular mortality and 2.3 servings/day for heart disease mortality, below which there was no significant associations with two outcomes. Considering the above-mentioned significant interaction between ultra-processed food consumption and sex, we performed sex-specific dose–response analyses (Figs. S1 and S2 for women and men, respectively). The nonlinear dose–response relationship of ultra-processed food consumption to overall cardiovascular and heart disease mortality was seen in both men and women (all *P*_nonlinearity_ < 0.001), but the thresholds for increased overall cardiovascular and heart disease mortality were lower in women than in men (women: 1.8 and 2.0 servings/day for overall cardiovascular and heart disease mortality, respectively; men: 4.1 and 3.3 servings/day for overall cardiovascular and heart disease mortality, respectively). No significant dose–response relationship was observed for cerebrovascular mortality in the whole study population and in men or women.

### Sensitivity analyses

The initial associations between ultra-processed food consumption and risks of death from CVD, heart disease, and cerebrovascular disease persisted in a large range of sensitivity analyses (Table S6).

### Contributions of and associations by ultra-processed food groups

**Fig. 4:**
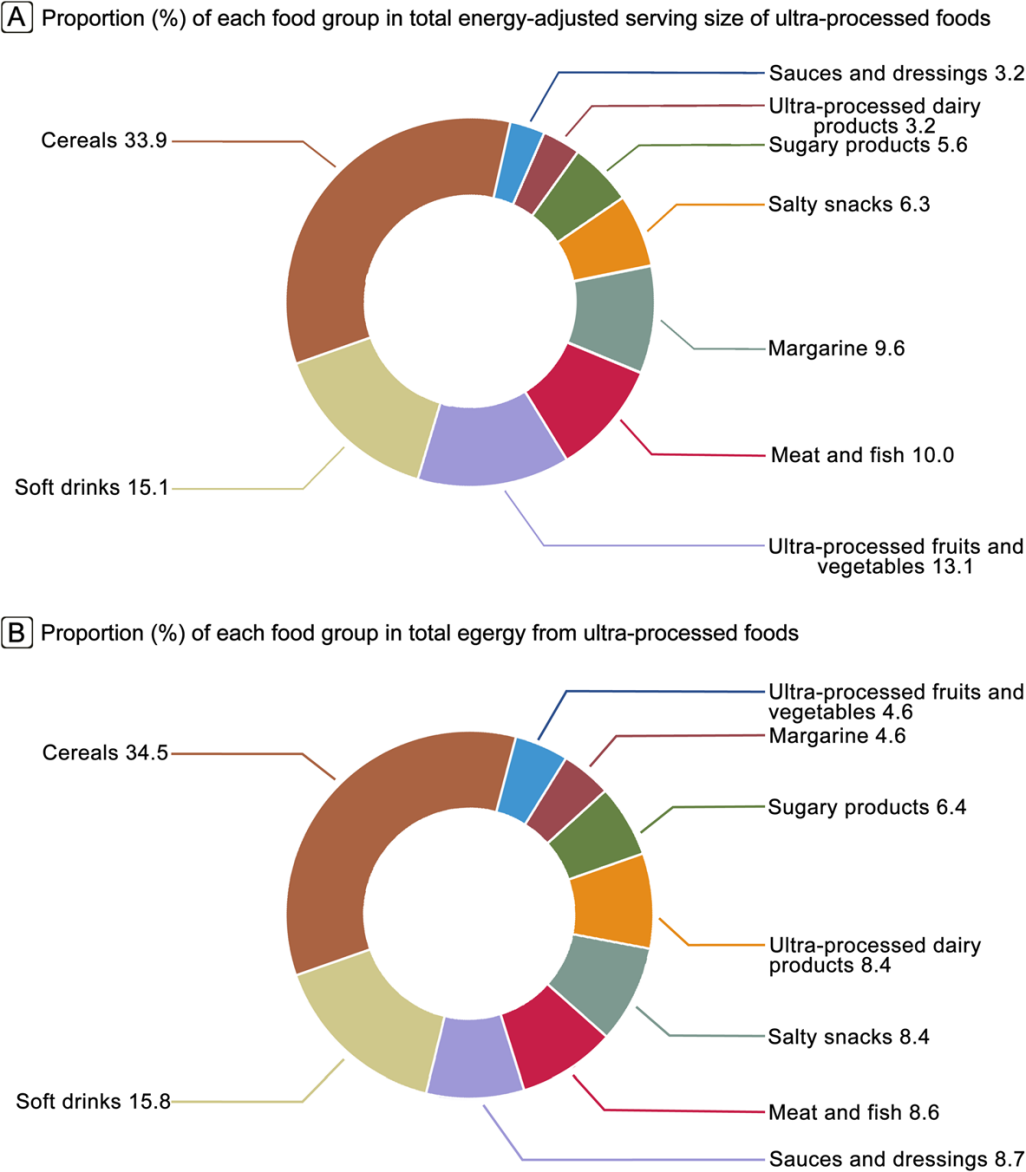
Proportion (%) of each food group in total amounts of ultra-processed foods in the whole study population

Main food groups contributing to total energy-adjusted serving size of ultra-processed foods were cereals (33.9%) followed by soft drinks (15.1%), ultra-processed fruits and vegetables (13.1%), and meat and fish (10.0%), while main food groups contributing to total energy from ultra-processed foods were cereals (34.5%) and soft drinks (15.8%) (Fig. 4). The highest vs. the lowest quintiles of consumption of soft drinks, meat and fish, salty snacks, and sugary products was found to be significantly associated with increased risks of overall cardiovascular and heart disease mortality (all *P*_trend_ < 0.05) (Table S7).

### Positive control outcome

A total of 19,586 all-cause deaths were documented during follow-up, with the overall mortality rate of 158.46 per 10,000 person-years. In the fully adjusted model, participants in the highest quintile of ultra-processed food consumption had a higher risk of all-cause mortality than those in the lowest quintile (HR _quintile 5 vs. 1_, 1.20; 95% CI, 1.14–1.26; *P*_trend_ < 0.001) (Table S8).

## Discussion

In this large prospective multicenter study with long-term follow-up, we revealed significant harmful associations of ultra-processed foods with risks of death from CVD and heart disease, with a threshold for harm above consumption of 2.4 servings/day for overall cardiovascular mortality and 2.3 servings/day for heart disease mortality. Sex-specific analyses further showed that these harmful associations were more pronounced in women than in men. No significant association was observed for cerebrovascular mortality.

### Interpretation and comparison with other studies

Several studies have examined the association of ultra-processed food consumption with all-cause mortality [15, 16, 23, 34], but few studies focus on cause-specific mortality that may be more biologically relevant to ultra-processed food consumption [15, 16]. In this study, we revealed a positive association of ultra-processed food consumption with cardiovascular mortality, which is inconsistent with previous studies on this topic [15, 16]. Specifically, with similar study design and methods, previous studies on this subject failed to detect a significant association [15, 16]. The inconsistency may result from the significant difference in the power. Of note, previous studies documented a small number of cardiovascular deaths (71 in Spanish study [15] and 648 in American study [16]), which results in the limited power. The inconsistency could be also due to the differences in sociodemographic characteristics of study population, considering that sociodemographic factors, such as age, race, and income, have been found to be associated with ultra-processed food consumption [8].

The harmful association of ultra-processed foods with cardiovascular mortality could be accounted by several factors. First, high ultra-processed food consumption will result in low consumption of non-ultra-processed foods [35], such as fresh fruits and vegetables, finally leading to the poor diet quality [36], which has been identified to be associated with increased cardiovascular mortality [37]. In fact, a recent prospective study found that the isocaloric replacement of ultra-processed foods with minimally processed or unprocessed foods would reduce mortality risk in theory [23], which supports the above-mentioned explanation, at least partly. Second, unfavorable nutritional composition of ultra-processed foods may be a key factor driving the observed associations. It has been found that ultra-processed food consumption is positively associated with added sugar intake and inversely associated with dietary fiber intake [38], both of which are shown to be predictive of cardiovascular mortality [39, 40]. Third, chemicals may transfer from packaging materials to food contents, some of which may have detrimental impacts on cardiometabolic health [41]. Indeed, a cross-sectional study showed that ultra-processed food consumption could increase exposure to phthalates (the synthetic chemicals widely used in food packaging) [42]; a recent Cochrane review further showed a significant association between exposure to phthalates and cardiometabolic risk factors [43]. Fourth, cosmetic food additives are frequently used in the production of ultra-processed foods [6], and some studies have reported their adverse effects on cardiovascular outcomes. For example, observational studies found that a high serum level of phosphate, a food additive commonly used in ultra-processed foods, was a risk factor for cardiovascular event [44, 45]. Additionally, a cell study indicated that long-term use of artificial sweeteners might exacerbate atherosclerosis [46]. Fifth, ultra-processed foods may contain some neo-formed contaminants formed during industrial processes that ultra-processed foods undergo, such as acrolein. Importantly, both in vitro and in vivo studies have suggested that acrolein has toxic effects on cardiovascular tissues [47]; observational studies further showed that exposure to acrolein was associated with an increased risk of CVD [47, 48].

### Explanations for sex difference in cardiovascular mortality

Interestingly, our study found that the increased risks of death from CVD and heart disease were more pronounced in women. Similarly, previous studies also observed that consumption of soft drinks and processed meat was positively associated with higher CVD incidence and mortality in women than in men [49–51]. In fact, sex difference in cardiovascular outcomes has long been recognized [52]. We propose the following explanations for this phenomenon.

On the one hand, biologically, as a result from the hormonal differences between men and women. As almost all women in our study had experienced menopause, thus one would not expect estrogen level difference between sexes to be a major driver for the sex-specific association of ultra-processed food consumption with cardiovascular mortality. Instead, testosterone may be involved in the relevant mechanisms. Indeed, testosterone signaling has been suggested to play an important role in maintaining cardiovascular health [53]; moreover, observational studies have observed inverse associations of endogenous testosterone levels with CVD incidence and mortality [54–56]. Thus, the fact that women have lower testosterone levels than men may explain, at least partly, the observed sex difference in cardiovascular mortality.

On the hand other, sex disparities in the prevention, diagnosis, and treatment of CVD should be considered. Generally, women are less likely to receive preventive guidance or therapy, be diagnosed appropriately, and be treated aggressively compared with their male counterparts [57, 58]. A registry study of 82,196 patients with acute coronary syndrome found that women received medical strategies and acute treatments for secondary prevention less frequently than did men [59]. Additionally, compared with men, women have a poorer adherence to the use of chronic medication [60]. In fact, even with a comparable adherence, women always benefit less from medication use than do men, considering the low enrollment of women in clinical trials of CVD [61], resulting in that current diagnostic and therapeutic methods primarily target men [62]. Therefore, sex disparities in access to health care possibly mediate sex difference in cardiovascular mortality owing to ultra-processed food consumption.

Importantly, we cannot exclude the possibility that sex difference in cardiovascular mortality observed in our study is a chance finding, although this phenomenon could be explained by the above-mentioned points. Hence, the stronger associations with cardiovascular mortality in women compared to men should be treated with caution, and needs to be confirmed by future studies.

### Limitations

Our study has several limitations. First, misclassification bias might occur when we categorized food items, as the DHQ did not always provide enough information for correct categorization of food items. However, this bias is nondifferential (because it was not expected to be associated with future cardiovascular mortality), and potentially biases risk estimates toward the null. Moreover, we observed an expected association between ultra-processed food consumption and positive control outcome, indicating the validity of our study methods. Second, though we adjusted for several confounders, our results may still be subject to residual confounding due to unrecognized or unmeasured confounders. Nevertheless, the E-value for overall cardiovascular mortality was 2.41 in our study setting, indicating that an unmeasured confounder with a HR ≥2.41 can explain away the observed association; the possibility of existing such an unmeasured confounder seems to be low, as the HR for the history of hypertension, a strong CVD risk factor, was only 1.52 in our study. Furthermore, as individuals with higher consumption of ultra-processed foods have been found to have a poorer overall diet both in our study and others [36], diet quality could be a potential mediator between ultra-processed food consumption and cardiovascular mortality. However, the fact that initial results persisted after we adjusted for several indicators of diet quality (Table S6), makes this unlikely. Third, food consumption was assessed once at baseline in our study. Considering that diet habits can change with time, the assessment of food consumption at one time point may result in non-differential bias. Nonetheless, one would not expect an adult’s diet habits to change drastically during several years; furthermore, it has been found that the approach using only baseline diet generally yields a weaker association than that using the cumulative averages [63]. Fourth, we chose serving size as an indicator for ultra-processed food consumption. However, this measure may not precisely reflect the contribution of ultra-processed foods in the diet, and possibly produces inaccurate results. Nonetheless, our initial results remained in analyses where ultra-processed food consumption was expressed as serving per day/kilogram body weight or % energy, which alleviates this concern to some extent. Fifth, our study could not examine the association of ultra-processed food consumption with CVD incidence, as this outcome was not available in the PLCO Cancer Screening Trial. Nonetheless, our study had revealed its harmful association with cardiovascular mortality, a primary outcome in the cardiology research. Finally, in the PLCO Cancer Screening Trial, 85.6% of study population were non-Hispanic white, around half were ever smokers, and around one-third were postgraduate or college graduate [64], all of which compromise the generalizability of our findings. Hence, future studies should validate our findings in other populations and settings.

## Conclusions

In the US population, high consumption of ultra-processed foods is associated with increased risks of death from CVD and heart disease. These harmful associations may be more pronounced in women than in men. Our findings suggest that reducing ultra-processed food consumption may be beneficial in reducing cardiovascular mortality, especially in women. However, these findings need to be confirmed in other populations and settings, considering the aforementioned limitations and the modest changes in mortality from CVD and heart disease even with large differences in ultra-processed food consumption. If confirmed, given the increasing dominance of ultra-processed foods in the global food system, limiting ultra-processed food consumption would represent an attractive strategy to reduce the global burden of CVD. Future studies should explore the relevant mechanisms and deepen the understanding of sex differences in the observed associations.

## Supplementary Information

**Additional file 1: Figure S1.** Nonlinear dose–response analyses on energy-adjusted ultra-processed food consumption and cardiovascular mortality in women. **Figure S2.** Nonlinear dose–response analyses on energy-adjusted ultra-processed food consumption and cardiovascular mortality in men. **Table S1.** Ultra-processed foods in each food group and energy values assigned to 65 food items of the diet history questionnaire. **Table S2.** Distribution of variables with missing data before and after multiple imputation. **Table S3.** Association between energy-adjusted ultra-processed food consumption (serving daily) and cardiovascular mortality in 67,823 participants with complete data. **Table S4.** Association between energy-adjusted ultra-processed food consumption (serving daily per kilogram body weight) and cardiovascular mortality. **Table S5.** Association between the proportion of energy from ultra-processed foods to total daily energy intake (% energy) and cardiovascular mortality. **Table S6.** Sensitivity analyses on the association between energy-adjusted ultra-processed food consumption (serving daily) and cardiovascular mortality. **Table S7.** Association between energy-adjusted ultra-processed food consumption by food group (serving daily) and cardiovascular mortality. **Table S8.** Association between energy-adjusted ultra-processed food consumption (serving daily) and all-cause mortality

## Data Availability

Original data used in this study are not freely available to the public because of US NCI’s data policy. These original data can be accessible upon the reasonable request and the final approval by US NCI.

## Abbreviations

HR: hazard ratio
CVD: cardiovascular disease
PLCO: Prostate, Lung, Colorectal, and Ovarian
DHQ: diet history questionnaire
BMI: body mass index
ICD: International Classification of Diseases
CI: confidence interval
