# Supplementary material for: Association of ultra-processed food consumption with cardiovascular mortality in the US population: long-term results from a large prospective multicenter study
Source: Int J Behav Nutr Phys Act. 2021 Feb 3;18:21. doi: 10.1186/s12966-021-01081-3 (PMC7860226; doi:10.1186/s12966-021-01081-3)
Supplement: Supplementary file 1 — Additional file 1: Figure S1. Nonlinear dose–response analyses on energy-adjusted ultra-processed food consumption and cardiovascular mortality in women. Figure S2. Nonlinear dose–response analyses on energy-adjusted ultra-processed food consumption and cardiovascular mortality in men. Table S1. Ultra-processed foods in each food group and energy values assigned to 65 food items of the diet history questionnaire. Table S2. Distribution of variables with missing data before and after multiple imputation. Table S3. Association between energy-adjusted ultra-processed food consumption (serving daily) and cardiovascular mortality in 67,823 participants with complete data. Table S4. Association between energy-adjusted ultra-processed food consumption (serving daily per kilogram body weight) and cardiovascular mortality. Table S5. Association between the proportion of energy from ultra-processed foods to total daily energy intake (% energy) and cardiovascular mortality. Table S6. Sensitivity analyses on the association between energy-adjusted ultra-processed food consumption (serving daily) and cardiovascular mortality. Table S7. Association between energy-adjusted ultra-processed food consumption by food group (serving daily) and cardiovascular mortality. Table S8. Association between energy-adjusted ultra-processed food consumption (serving daily) and all-cause mortality [file 12966_2021_1081_MOESM1_ESM.doc]

ADDITIONAL FILES

**Association of ultra-processed food consumption with cardiovascular mortality in the US population: long-term results from a large prospective multicenter study**

Guo-Chao Zhong, Hai-Tao Gu, Yang Peng, Kang Wang, You-Qi-Le Wu, Tian-Yang Hu, Feng-Chuang Jing, and Fa-Bao Hao


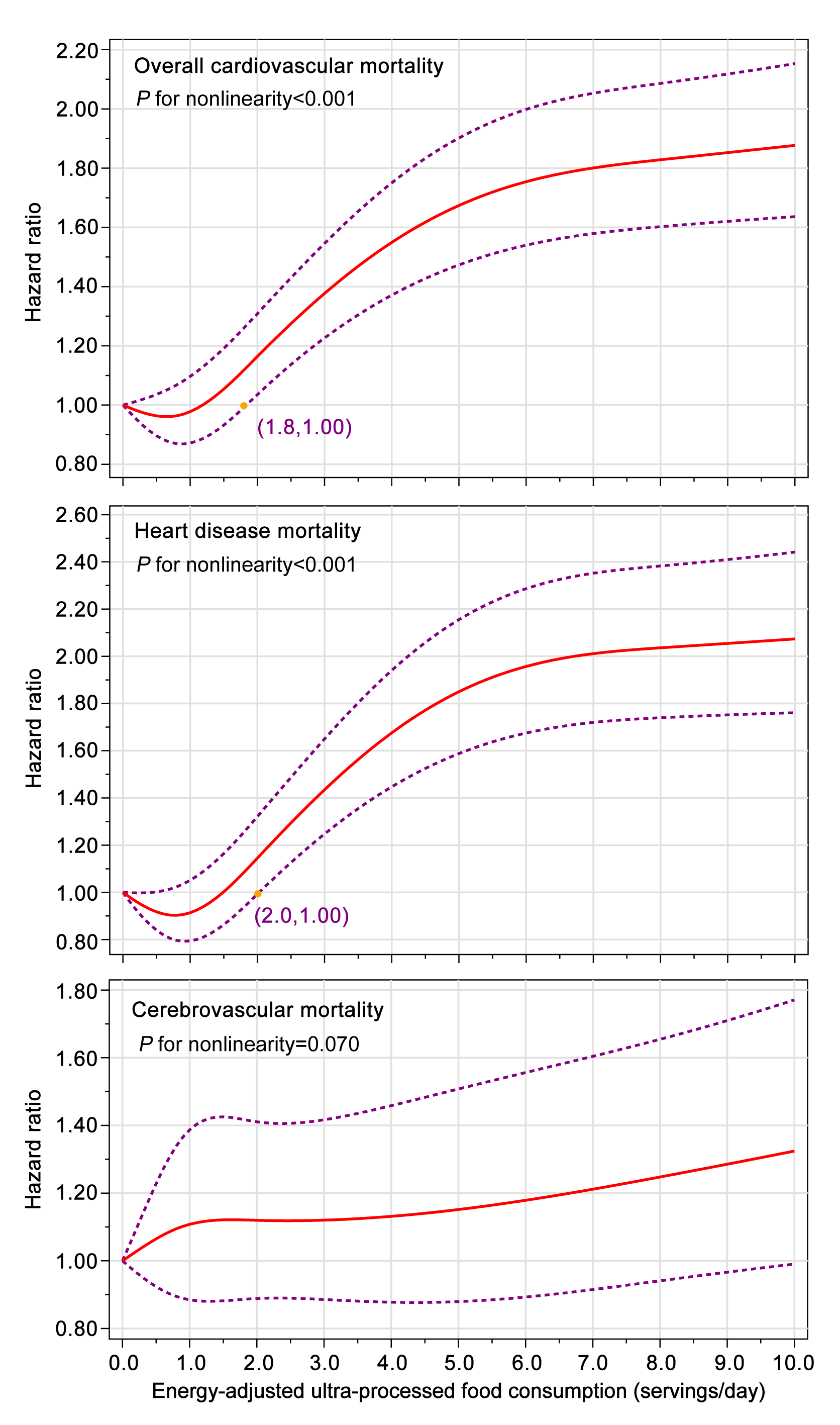


**Figure S1** Nonlinear dose–response analyses on energy-adjusted ultra-processed food consumption and cardiovascular mortality in women. Hazard ratio was adjusted for age, race, educational level, marital status, study center, aspirin use, history of hypertension, history of diabetes, smoking status, alcohol consumption, body mass index, physical activity level, and energy intake from diet. The red solid line represents the fitted nonlinear trend, and the purple short-dash line represents corresponding 95% confidence interval.


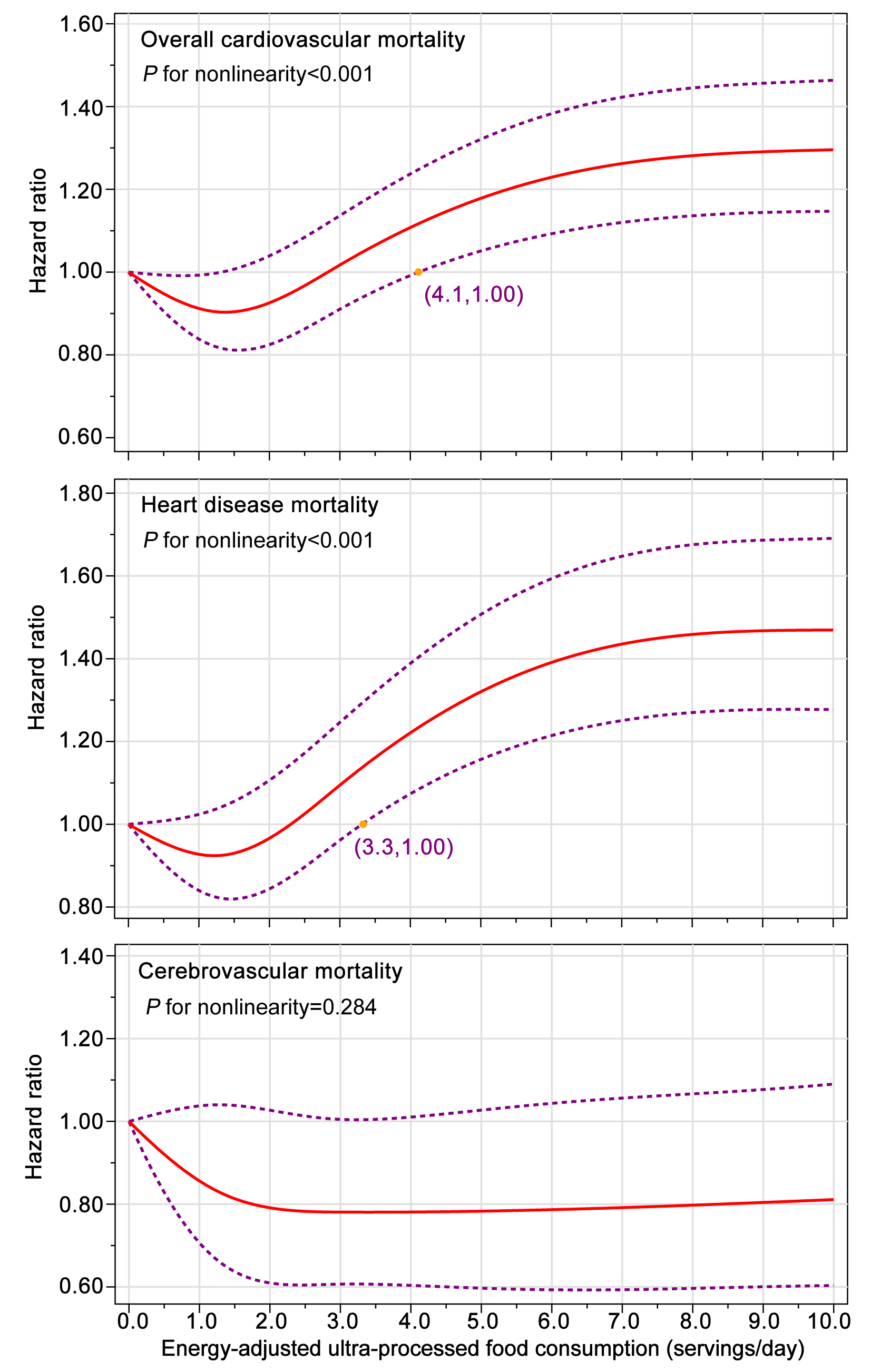


**Figure S2** Nonlinear dose–response analyses on energy-adjusted ultra-processed food consumption and cardiovascular mortality in men. Hazard ratio was adjusted for age, race, educational level, marital status, study center, aspirin use, history of hypertension, history of diabetes, smoking status, alcohol consumption, body mass index, physical activity level, and energy intake from diet. The red solid line represents the fitted nonlinear trend, and the purple short-dash line represents corresponding 95% confidence interval.

| **Table S1.** Ultra-processed foods in each food group and energy values assigned to 65 food items of the diet history questionnaire | | |
| --- | --- | --- |
| Food group | Ultra-processed foods | Energy per 100 g (kcal) |
| Soft Drinks | Soft drinks, diet/caffeinated | 49 |
|  | Soft drinks, regular/caffeinated | 49 |
|  | Soft drinks, diet/decaffeinated | 40 |
|  | Soft drinks, regular/decaffeinated | 40 |
| Cereals | Hot dogs, turkey/low fat | 127 |
|  | Hot dogs, regular | 332 |
|  | English muffins and bagels | 227 |
|  | White bread/rolls | 266 |
|  | Corn bread/muffins | 305 |
|  | Biscuits | 324 |
|  | Quick breads | 297 |
|  | Donuts, sweet rolls, Danishes, and pop tarts | 321 |
|  | Pancakes, waffles, and French toast | 270 |
|  | Ready-to-eat cereal, highly fortified | 374 |
|  | Ready-to-eat cereal, other | 374 |
|  | Cookies and brownies | 405 |
|  | Cakes, low fat | 283 |
|  | Cakes, regular | 389 |
|  | Pies, cream/custard/other | 210 |
|  | Pies, fruit | 209 |
|  | Pies, pecan | 407 |
|  | Pies, pumpkin/sweet potato | 260 |
|  | Lasagna, ravioli, shells | 177 |
|  | Macaroni and cheese | 221 |
|  | Pizza without meat | 233 |
| Ultra-processed fruits and vegetables | Fried potatoes | 196 |
|  | Potato salad | 173 |
|  | Fruit drinks, diet | 1 |
|  | Fruit drinks, regular | 10 |
| Ultra-processed dairy products | Sour cream, low fat | 181 |
|  | Sour cream, regular | 198 |
|  | Cream cheese, low fat | 201 |
|  | Cream cheese, regular | 350 |
|  | Ice cream/ice milk, low fat | 207 |
|  | Regular ice cream | 216 |
|  | Frozen yogurt, ices, and sorbet | 72 |
|  | Creamed soups | 46 |
| Meat and fish | Restaurant or industrial hamburgers | 244 |
|  | Fried chicken, dark meat/no skin | 164 |
|  | Fried chicken, dark meat/with skin | 251 |
|  | Fried chicken, white meat/no skin | 164 |
|  | Fried chicken, white meat/with skin | 251 |
|  | Fried fish, fat added | 237 |
|  | Pizza with meat | 277 |
| Sauces and dressings | Gravy | 53 |
|  | Salad dressing, low fat on salad and vegetables | 160 |
|  | Salad dressing, nonfat on salad and vegetables | 107 |
|  | Salad dressing, regular on salad and vegetables | 430 |
|  | Mayonnaise, diet on salad | 250 |
|  | Mayonnaise, fat free on salad | 64 |
|  | Mayonnaise, regular on salad | 680 |
|  | Mayonnaise, diet on sandwich | 250 |
|  | Mayonnaise, fat free on sandwich | 64 |
|  | Mayonnaise, regular on sandwich | 680 |
| Salty snacks | Crackers | 430 |
|  | Potato/corn/other chips | 532 |
|  | Potato/corn/other chips-low fat | 482 |
|  | Popcorn | 530 |
| Sugary products | Candy, chocolate | 462 |
|  | Candy, not chocolate | 394 |
|  | Saccharine in coffee and tea | 360 |
| Margarine | Margarine, diet | 533 |
|  | Margarine, fat free | 330 |
|  | Margarine, regular | 533 |
|  | Margarine, diet on bread | 533 |

| **Table S2** Distribution of variables with missing data before and after multiple imputation | | | |
| --- | --- | --- | --- |
| Variable | Before multiple imputation | After multiple imputation | Number (%) with missing data |
| Physical activity (min/week) ^a^ | 125.5 ± 122.5 | 125.4 ± 124.2 | 22617 (24.61) |
| Marital status | | | |
| Married | 54007 (79.6) | 72146 (78.5) | 171 (0.19) |
| Widowed | 5066 (7.5) | 7343 (8.0) |
| Divorced | 6202 (9.1) | 8804 (9.6) |
| Separated | 471 (0.7) | 693 (0.8) |
| Never married | 2077 (3.1) | 2905 (3.2) |
| History of diabetes | | | |
| Yes | 3317 (4.9) | 5324 (5.8) | 524 (0.57) |
| No | 64506 (95.1) | 86567 (94.2) |
| History of hypertension | | | |
| Yes | 19701 (29.0) | 27939 (30.4) | 508 (0.55) |
| No | 48122 (71.0) | 63952 (69.6) |
| Educational level | | | |
| College below | 41640 (61.4) | 58140 (63.3) | 181 (0.20) |
| College graduate | 12547 (18.5) | 16367 (17.8) |
| Postgraduate | 13636 (20.1) | 17384 (18.9) |
| Smoking status | | | |
| Current | 5513 (8.1) | 8411 (9.2) | 20 (0.02) |
| Former | 28376 (41.8) | 38379 (41.8) |
| Never | 33934 (50.0) | 45101 (49.1) |
| Body mass index (kg/m^2^) | | | |
| <18.5 | 427 (0.6) | 618 (0.7) | 1211 (1.32) |
| 18.5–24.9 | 23598 (34.8) | 31242 (34.5) |
| 25.0–30.0 | 28889 (42.6) | 38387 (42.3) |
| >30 | 14909 (22.0) | 20433 (22.5) |

Values are mean (standard deviation) or counts (percentage) as indicated.

^a^ Total time of moderate to vigorous physical activities per week.

| **Table S3.** Association between energy-adjusted ultra-processed food consumption (daily serving) and cardiovascular mortality in 67823 participants with complete data | | | | | | |
| --- | --- | --- | --- | --- | --- | --- |
| Causes of mortality | Quintiles of energy-adjusted ultra-processed food consumption, range (mean), servings/day | | | | | *P*_trend_ |
|  | <0.5 (0.1) | 0.5–<1.2 (0.8) | 1.2–<2.2 (1.6) | 2.2–≤4.0 (3.0) | >4.0 (8.1) |  |
| Cardiovascular disease | | | | | | |
| No. of deaths | 523 | 523 | 545 | 617 | 722 |  |
| Death rate ^a^ | 28.74 | 28.39 | 29.51 | 33.79 | 40.31 |  |
| Model 1 ^b^ | 1.00 (reference) | 0.98 (0.87, 1.11) | 1.03 (0.91, 1.17) | 1.29 (1.14, 1.45 | 1.71 (1.52, 1.92) | <0.001 |
| Model 2 ^c^ | 1.00 (reference) | 0.95 (0.84, 1.08) | 1.02 (0.90, 1.16) | 1.24 (1.10, 1.40) | 1.56 (1.38, 1.78) | <0.001 |
| Heart disease | | | | | | |
| No. of deaths | 346 | 353 | 375 | 473 | 592 |  |
| Death rate ^a^ | 19.02 | 19.16 | 20.30 | 25.90 | 33.05 |  |
| Model 1 ^b^ | 1.00 (reference) | 1.00 (0.86, 1.16) | 1.07 (0.92, 1.24) | 1.47 (1.27, 1.69) | 2.05 (1.79, 2.36) | <0.001 |
| Model 2 ^c^ | 1.00 (reference) | 0.96 (0.83, 1.12) | 1.04 (0.89, 1.20) | 1.38 (1.19, 1.60) | 1.81 (1.56, 2.10) | <0.001 |
| Cerebrovascular disease | | | | | | |
| No. of deaths | 145 | 130 | 129 | 105 | 94 |  |
| Death rate ^a^ | 7.97 | 7.06 | 6.98 | 5.75 | 5.25 |  |
| Model 1 ^b^ | 1.00 (reference) | 0.88 (0.69, 1.11) | 0.90 (0.71, 1.15) | 0.82 (0.64, 1.07) | 0.88 (0.67, 1.15) | 0.4522 |
| Model 2 ^c^ | 1.00 (reference) | 0.87 (0.69, 1.12) | 0.96 (0.75, 1.23) | 0.89 (0.68, 1.16) | 0.94 (0.70, 1.25) | 0.8797 |

Values are hazard ratios (95% confidence intervals).

^a^ Crude death rate per 10000 person-years.

^b^ Adjusted for age (years), sex (male, female), race (non-Hispanic white, non-Hispanic black, Hispanic, others), educational level (college below, college graduate, postgraduate), marital status (married, widowed, divorced, separated, never married), and study center (10 categories).

^c^ Adjusted for model 1 plus aspirin use (yes, no), history of hypertension (yes, no), history of diabetes (yes, no), smoking status (current, former, never), alcohol consumption (g/day), body mass index (<18.5, 18.5–24.9, 25.0–30.0, >30.0), physical activity (min/week), and energy intake from diet (kcal/day).

| **Table S4.** Association between energy-adjusted ultra-processed food consumption (serving per day/kilogram body weight) and cardiovascular mortality | | | | | | |
| --- | --- | --- | --- | --- | --- | --- |
| Causes of mortality | Quintile 1 | Quintile 2 | Quintile 3 | Quintile 4 | Quintile 5 | *P*_trend_ |
| Cardiovascular disease | | | | | | |
| No. of deaths | 1022 | 1013 | 994 | 1138 | 1266 |  |
| Death rate ^a^ | 41.64 | 41.35 | 40.53 | 46.40 | 51.75 |  |
| Model 1 ^b^ | 1.00 (reference) | 1.00 (0.91, 1.09) | 0.99 (0.90, 1.08) | 1.20 (1.10, 1.31) | 1.50 (1.37, 1.63) | <0.001 |
| Model 2 ^c^ | 1.00 (reference) | 0.99 (0.87, 1.11) | 0.96 (0.85, 1.09) | 1.23 (1.09, 1.39) | 1.51 (1.33, 1.71) | <0.001 |
| Heart disease | | | | | | |
| No. of deaths | 700 | 690 | 687 | 863 | 1006 |  |
| Death rate ^a^ | 28.52 | 28.17 | 28.01 | 35.19 | 41.13 |  |
| Model 1 ^b^ | 1.00 (reference) | 0.99 (0.89, 1.10) | 0.98 (0.89, 1.09) | 1.30 (1.18, 1.44) | 1.68 (1.53, 1.86) | <0.001 |
| Model 2 ^c^ | 1.00 (reference) | 0.99 (0.86, 1.15) | 0.98 (0.84, 1.14) | 1.37 (1.19, 1.59) | 1.73 (1.50, 2.00) | <0.001 |
| Cerebrovascular disease | | | | | | |
| No. of deaths | 261 | 235 | 228 | 205 | 186 |  |
| Death rate ^a^ | 10.64 | 9.59 | 9.30 | 8.36 | 7.60 |  |
| Model 1 ^b^ | 1.00 (reference) | 0.91 (0.76, 1.09) | 0.91 (0.76, 1.09) | 0.89 (0.74, 1.08) | 0.94 (0.77, 1.14) | 0.701 |
| Model 2 ^c^ | 1.00 (reference) | 0.90 (0.71, 1.14) | 0.91 (0.71, 1.16) | 0.82 (0.62, 1.07) | 0.95 (0.73, 1.26) | 0.909 |

Values are hazard ratios (95% confidence intervals).

^a^ Crude death rate per 10000 person-years.

^b^ Adjusted for age (years), sex (male, female), race (non-Hispanic white, non-Hispanic black, Hispanic, others), educational level (college below, college graduate, postgraduate), marital status (married, widowed, divorced, separated, never married), and study center (10 categories).

^c^ Adjusted for model 1 plus history of aspirin use (yes, no), hypertension (yes, no), history of diabetes (yes, no), smoking status (current, former, never), alcohol consumption (g/day), body mass index (<18.5, 18.5–24.9, 25.0–30.0, >30.0), physical activity (min/week), and energy intake from diet (kcal/day).

| **Table S5.** Association between the proportion of energy from ultra-processed foods to total daily energy intake (% energy) and cardiovascular mortality | | | | | | |
| --- | --- | --- | --- | --- | --- | --- |
| Causes of mortality | Quintiles of ultra-processed food consumption, range (mean), % energy | | | | | *P*_trend_ |
|  | <22.8 (17.0) | 22.8–<30.1 (26.6) | 30.1–<37.0 (33.5) | 37.0–≤41.2 (44.0) | >46.1 (59.4) |  |
| Cardiovascular disease | | | | | | |
| No. of deaths | 1054 | 982 | 1082 | 1127 | 1245 |  |
| Death rate ^a^ | 42.67 | 39.75 | 43.70 | 45.56 | 50.38 |  |
| Model 1 ^b^ | 1.00 (reference) | 0.94 (0.86, 1.03) | 1.02 (0.93, 1.11) | 1.08 (0.99, 1.18) | 1.31 (1.20, 1.42) | <0.001 |
| Model 2 ^c^ | 1.00 (reference) | 0.91 (0.81, 1.03) | 1.00 (0.89, 1.13) | 1.04 (0.92, 1.17) | 1.21 (1.07, 1.37) | <0.001 |
| Heart disease | | | | | | |
| No. of deaths | 722 | 683 | 793 | 838 | 949 |  |
| Death rate ^a^ | 29.23 | 27.65 | 32.03 | 33.88 | 38.40 |  |
| Model 1 ^b^ | 1.00 (reference) | 0.95 (0.86, 1.06) | 1.09 (0.98, 1.20) | 1.16 (1.05, 1.29) | 1.43 (1.29, 1.58) | <0.001 |
| Model 2 ^c^ | 1.00 (reference) | 0.91 (0.79, 1.06) | 1.07 (0.92, 1.23) | 1.14 (0.98, 1.31) | 1.34 (1.16, 1.55) | <0.001 |
| Cerebrovascular disease | | | | | | |
| No. of deaths | 257 | 234 | 208 | 210 | 217 |  |
| Death rate ^a^ | 10.40 | 9.47 | 8.40 | 8.49 | 8.78 |  |
| Model 1 ^b^ | 1.00 (reference) | 0.92 (0.77, 1.10) | 0.81 (0.67, 0.98) | 0.85 (0.71, 1.03) | 0.99 (0.83, 1.20) | 0.824 |
| Model 2 ^c^ | 1.00 (reference) | 0.85 (0.67, 1.09) | 0.74 (0.57, 0.96) | 0.79 (0.61, 1.02) | 0.87 (0.67, 1.13) | 0.319 |

Values are hazard ratios (95% confidence intervals).

^a^ Crude death rate per 10000 person-years.

^b^ Adjusted for age (years), sex (male, female), race (non-Hispanic white, non-Hispanic black, Hispanic, others), educational level (college below, college graduate, postgraduate), marital status (married, widowed, divorced, separated, never married), and study center (10 categories).

^c^ Adjusted for model 1 plus aspirin use (yes, no), history of hypertension (yes, no), history of diabetes (yes, no), smoking status (current, former, never), alcohol consumption (g/day), body mass index (<18.5, 18.5–24.9, 25.0–30.0, >30.0), and physical activity (min/week).

| **Table S6.** Sensitivity analyses on the association between energy-adjusted ultra-processed food consumption (daily serving) and cardiovascular mortality | | | | | | |
| --- | --- | --- | --- | --- | --- | --- |
| Categories | Overall cardiovascular mortality | | Heart disease mortality | | Cerebrovascular disease mortality | |
|  | HR_q5 vs. 1_ (95% CI) ^a^ | *P*_trend_ | HR_q5 vs. 1_ (95% CI) ^a^ | *P*_trend_ | HR_q5 vs. 1_ (95% CI) ^a^ | *P*_trend_ |
| Excluding deaths observed within the first five years of follow-up | 1.51 (1.37, 1.66) | <0.001 | 1.68 (1.51, 1.88) | <0.001 | 0.99 (0.79, 1.22) | 0.900 |
| Excluding subjects with extreme values of energy intake ^b^ | 1.49 (1.36, 1.64) | <0.001 | 1.67 (1.49, 1.86) | <0.001 | 0.96 (0.77, 1.19) | 0.861 |
| Including subjects with history of cancer at baseline | 1.53 (1.39, 1.67) | <0.001 | 1.71 (1.52, 1.89) | <0.001 | 0.95 (0.76, 1.18) | 0.678 |
| Including subjects with history of heart attack or stroke at baseline ^c^ | 1.43 (1.31, 1.54) | <0.001 | 1.68 (1.50, 1.84) | <0.001 | 0.94 (0.81, 1.11) | 0.701 |
| Repeating analysis with competing risk regression model ^d^ | 1.53 (1.40, 1.70) | <0.001 | 1.69 (1.50, 1.89) | <0.001 | 0.96 (0.78, 1.20) | 0.686 |
| Adjustment for propensity score on unadjusted model | 1.51 (1.39, 1.64) | <0.001 | 1.77 (1.61, 1.95) | <0.001 | 0.82 (0.68, 1.00) | 0.050 |
| Additional adjustment on model 2 ^e^ | | | | | | |
| Healthy Eating Index-2005 | 1.48 (1.35, 1.63) | <0.001 | 1.67 (1.49, 1.86) | <0.001 | 0.94 (0.75, 1.16) | 0.687 |
| Intakes of sodium, added sugars, and saturated fatty acids | 1.48 (1.34, 1.63) | <0.001 | 1.65 (1.47, 1.85) | <0.001 | 0.93 (0.74, 1.17) | 0.622 |
| Consumption of red meat, processed meat, whole grain, fruit, vegetable, dietary fiber, and dairy | 1.49 (1.35, 1.64) | <0.001 | 1.66 (1.48, 1.86) | <0.001 | 0.96 (0.77, 1.20) | 0.802 |

Values are hazard ratios (95% confidence intervals) unless otherwise specified.

^a^ The risk estimates were adjusted for the following variables unless otherwise specified: age (years), sex (male, female), race (non-Hispanic white, non-Hispanic black, Hispanic, others), educational level (college below, college graduate, postgraduate), marital status (married, widowed, divorced, separated, never married), study center (10 categories), aspirin use (yes, no), history of hypertension (yes, no), history of diabetes (yes, no), smoking status (current, former, never), alcohol consumption (g/day), body mass index (<18.5, 18.5–24.9, 25.0–30.0, >30.0), physical activity (min/week), and energy intake from diet (kcal/day).

^b^ Extreme values of energy intake are defined as <800 or >4000 kcal/d for men and <500 or >3500 kcal/d for women.

^c^ The risk estimates were additionally adjusted for history of heart attack (yes, no) and history of stroke (yes, no).

^d^ The results were expressed as subdistribution hazard ratio and 95% confidence interval.

^e^ All variables were treated as the continuous variable in multivariable Cox regression.

| **Table S7.** Association between energy-adjusted ultra-processed food consumption by food group (daily serving) and cardiovascular mortality | | | | | | |
| --- | --- | --- | --- | --- | --- | --- |
| Food groups | Multivariable-adjusted hazard ratios (95% confidence interval) ^a^ | | | | | *P*_trend_ |
|  | Quintile 1 | Quintile 2 | Quintile 3 | Quintile 4 | Quintile 5 |  |
| Overall cardiovascular mortality | | | | | | |
| Cereals | 1.00 (reference) | 0.94 (0.86, 1.03) | 0.95 (0.87, 1.04) | 0.93 (0.85, 1.02) | 1.06 (0.96, 1.18) | 0.085 |
| Soft Drinks | 1.00 (reference) | 0.90 (0.82, 0.98) | 0.89 (0.81, 0.97) | 0.98 (0.90, 1.07) | 1.27 (1.16, 1.38) | <0.001 |
| Sauces and dressings | 1.00 (reference) | 0.99 (0.91, 1.08) | 1.00 (0.91, 1.08) | 0.92 (0.84, 1.00) | 1.10 (1.01, 1.23) | 0.054 |
| Meat and fish | 1.00 (reference) | 1.09 (1.00, 1.19) | 1.02 (0.93, 1.12) | 1.10 (1.01, 1.21) | 1.14 (1.03, 1.26) | 0.017 |
| Salty snacks | 1.00 (reference) | 0.97 (0.89, 1.05) | 1.00 (0.92, 1.09) | 0.90 (0.82, 0.98) | 1.10 (1.01, 1.20) | 0.020 |
| Ultra-processed dairy products | 1.00 (reference) | 0.98 (0.90, 1.06) | 0.91 (0.84, 1.00) | 0.89 (0.82, 0.98) | 0.95 (0.87, 1.04) | 0.467 |
| Margarine | 1.00 (reference) | 1.01 (0.93, 1.11) | 1.03 (0.94, 1.12) | 1.02 (0.93, 1.11) | 0.98 (0.89, 1.07) | 0.389 |
| Sugary products | 1.00 (reference) | 0.96 (0.88, 1.04) | 0.79 (0.72, 0.87) | 0.99 (0.91, 1.08) | 1.19 (1.10, 1.30 | <0.001 |
| Ultra-processed fruits and vegetables | 1.00 (reference) | 0.93 (0.86, 1.02) | 0.97 (0.89, 1.06) | 0.97 (0.89, 1.05) | 1.04 (0.95, 1.13) | 0.075 |
| Heart disease mortality | | | | | | |
| Cereals | 1.00 (reference) | 0.93 (0.84, 1.04) | 0.94 (0.85, 1.05) | 0.91 (0.82, 1.02) | 1.05 (0.93, 1.18) | 0.197 |
| Soft Drinks | 1.00 (reference) | 0.87 (0.78, 0.96) | 0.87 (0.79, 0.97) | 1.01 (0.91, 1.12) | 1.37 (1.24, 1.52) | <0.001 |
| Sauces and dressings | 1.00 (reference) | 1.01 (0.92, 1.11) | 0.96 (0.86, 1.06) | 0.94 (0.85, 1.04) | 1.11 (1.00, 1.23) | 0.065 |
| Meat and fish | 1.00 (reference) | 1.12 (1.01, 1.25) | 1.09 (0.98, 1.21) | 1.15 (1.04, 1.29) | 1.17 (1.04, 1.31) | 0.044 |
| Salty snacks | 1.00 (reference) | 0.97 (0.88, 1.07) | 1.02 (0.92, 1.12) | 0.92 (0.83, 1.02) | 1.11 (1.01, 1.24) | 0.023 |
| Ultra-processed dairy products | 1.00 (reference) | 0.97 (0.88, 1.07) | 0.89 (0.80, 0.99) | 0.86 (0.78, 0.96) | 0.95 (0.86, 1.06) | 0.683 |
| Margarine | 1.00 (reference) | 1.05 (0.94, 1.16) | 1.04 (0.94, 1.15) | 1.04 (0.94, 1.15) | 1.02 (0.92, 1.13) | 0.984 |
| Sugary products | 1.00 (reference) | 0.97 (0.88, 1.07) | 0.80 (0.72, 0.89) | 1.01 (0.92, 1.12) | 1.21 (1.10, 1.34) | <0.001 |
| Ultra-processed fruits and vegetables | 1.00 (reference) | 0.95 (0.86, 1.05) | 1.04 (0.94, 1.16) | 1.04 (0.94, 1.15) | 1.11 (1.00, 1.23) | 0.013 |
| Cerebrovascular mortality | | | | | | |
| Cereals | 1.00 (reference) | 1.02 (0.85, 1.23) | 0.98 (0.81, 1.19) | 0.98 (0.80, 1.20) | 1.19 (0.95, 1.50) | 0.138 |
| Soft Drinks | 1.00 (reference) | 0.99 (0.83, 1.18) | 0.92 (0.76, 1.11) | 0.86 (0.71, 1.05) | 0.97 (0.79, 1.18) | 0.816 |
| Sauces and dressings | 1.00 (reference) | 0.89 (0.74, 1.07) | 0.98 (0.82, 1.17) | 0.87 (0.73, 1.04) | 1.03 (0.85, 1.25) | 0.779 |
| Meat and fish | 1.00 (reference) | 1.00 (0.83, 1.19) | 0.84 (0.70, 1.02) | 0.92 (0.76, 1.13) | 1.08 (0.86, 1.34) | 0.372 |
| Salty snacks | 1.00 (reference) | 0.99 (0.83, 1.18) | 0.97 (0.81, 1.17) | 0.85 (0.70, 1.04) | 1.00 (0.82, 1.23) | 0.867 |
| Ultra-processed dairy products | 1.00 (reference) | 1.03 (0.85, 1.24) | 1.02 (0.84, 1.23) | 1.02 (0.84, 1.25) | 1.01 (0.82, 1.24) | 0.965 |
| Margarine | 1.00 (reference) | 0.90 (0.74, 1.09) | 1.02 (0.85, 1.22) | 0.91 (0.75, 1.10) | 0.81 (0.67, 0.99) | 0.043 |
| Sugary products | 1.00 (reference) | 0.91 (0.76, 1.09) | 0.73 (0.60, 0.89) | 0.90 (0.75, 1.09) | 1.18 (0.98, 1.42) | 0.001 |
| Ultra-processed fruits and vegetables | 1.00 (reference) | 0.84 (0.70, 1.00) | 0.79 (0.66, 0.95) | 0.74 (0.61, 0.90) | 0.85 (0.70, 1.03) | 0.513 |

^a^ Adjusted for age (years), sex (male, female), race (non-Hispanic white, non-Hispanic black, Hispanic, others), educational level (college below, college graduate, postgraduate), marital status (married, widowed, divorced, separated, never married), study center (10 categories), aspirin use (yes, no), history of hypertension (yes, no), history of diabetes (yes, no), smoking status (current, former, never), alcohol consumption (g/day), body mass index (<18.5, 18.5–24.9, 25.0–30.0, >30.0), physical activity (min/week), and energy intake from diet (kcal/day). All ultra-processed food groups were mutually adjusted, with the consumption of each food group treated as the continuous variable in regression models.

| **Table S8.** Association between energy-adjusted ultra-processed food consumption (daily serving) and all-cause mortality | | | | | | |
| --- | --- | --- | --- | --- | --- | --- |
|  | Quintiles of energy-adjusted ultra-processed food consumption, range (mean), servings/day | | | | | *P*_trend_ |
|  | <0.5 (0.1) | 0.5–<1.2 (0.8) | 1.2–<2.1 (1.6) | 2.1–≤4.0 (3.0) | >4.1 (8.2) |  |
| No. of deaths | 3769 | 3862 | 3832 | 3931 | 4192 |  |
| Death rate ^a^ | 152.32 | 156.35 | 154.87 | 158.83 | 169.94 |  |
| Model 1 ^b^ | 1.00 (reference) | 1.01 (0.97, 1.06) | 1.01 (0.96, 1.05) | 1.11 (1.06, 1.16) | 1.28 (1.23, 1.34) | <0.001 |
| Model 2 ^c^ | 1.00 (reference) | 1.00 (0.96, 1.05) | 1.00 (0.95, 1.05) | 1.07 (1.02, 1.13) | 1.20 (1.14, 1.26) | <0.001 |

Values are hazard ratios (95% confidence intervals).

^a^ Crude death rate per 10000 person-years.

^b^ Adjusted for age (years), sex (male, female), race (non-Hispanic white, non-Hispanic black, Hispanic, others), educational level (college below, college graduate, postgraduate), marital status (married, widowed, divorced, separated, never married), and study center (10 categories).

^c^ Adjusted for model 1 plus history of hypertension (yes, no), aspirin use (yes, no), history of diabetes (yes, no), family history of cancer (yes, no), smoking status (current, former, never), alcohol consumption (g/day), body mass index (<18.5, 18.5–24.9, 25.0–30.0, >30.0), physical activity (min/week), and energy intake from diet (kcal/day).
